# P05.67. A narrative review of research on Ayurveda

**DOI:** 10.1186/1472-6882-12-S1-P427

**Published:** 2012-06-12

**Authors:** R Manohar, S Eranezhath, A Mahapatra, S Manohar R

**Affiliations:** 1 The Ayurvedic Trust, Coimbatore, India

## Purpose

There is limited access to published research in the field of Ayurveda. Researchers find themselves groping in the dark to make an assessment of prior research work. A narrative review of prior research by screening published research papers will lay the foundation for identifying strengths and gaps in the evidence base that is available to vouchsafe the safety and efficacy of Ayurvedic interventions. It will also facilitate systematic reviews and meta analyses in the future.

## Methods

More than 700 research journals on Ayurveda were accessed through electronic databases like Pubmed and DHARA and many journals were also hand search to compile nearly 8000 citations on Ayurvedic research. The research papers were screened and classified on the basis of the nature of submission, the research approach, quality of research, study design, disease conditions, formulations, interventions and species used.

## Results

A summarized and classified overview of prior research on Ayurveda could be sketched on the basis of the comprehensive analysis. Issues in quality of research and research design could be identified as well as the good and bad practices in research publication. The key diseases for which evidence is available for recipes and clinical interventions in Ayurveda could also be identified with an understanding of the level of evidence and the research gaps.

## Conclusion

Nearly half of the published research papers on Ayurveda are review papers. The majority of original research work is in the area of preclinical studies and drug standardization. Clinical research work is comparatively less and there is scarcity of research designs and studies that address Ayurveda as a whole system. However, clinical trials outnumber case studies and case reports. An analysis of the preclinical and clinical work done in the field of Ayurveda gives important leads to identify priorities for future research on Ayurveda.

